# Lipids and carotid plaque in the Northern Manhattan Study (NOMAS)

**DOI:** 10.1186/1471-2261-9-55

**Published:** 2009-12-22

**Authors:** Hannah Gardener, David Della Morte, Mitchell SV Elkind, Ralph L Sacco, Tatjana Rundek

**Affiliations:** 1 Department of Neurology, Miller School of Medicine, University of Miami, USA; 2 Division of Stroke, Department of Neurology, Columbia University, New York, NY, USA; 3 Department of Epidemiology, Miller School of Medicine, University of Miami, Miami, FL, USA; 4 Department of Human Genetics, Miller School of Medicine, University of Miami, Miami, FL, USA

## Abstract

**Background:**

Lipids, particularly low-density (LDL) and high-density (HDL) lipoproteins, are associated with increased risk of stroke and cardiovascular disease, probably due to atherosclerosis. The objective of this cross-sectional analysis was to investigate the relation between blood lipids and carotid plaque.

**Methods:**

As part of a prospective population-based study to determine the incidence and risk factors of stroke in a multiethnic population, we evaluated 1804 participants with lipid measurements and B-mode ultrasound of carotid arteries (mean age 69 +/- 10 years; 40% men; 51% Hispanic, 26% black, 23% white). The association between lipid parameters and carotid plaque was analyzed by multiple logistic regression.

**Results:**

Plaque was present in 61% of participants. Mean total cholesterol was 202 +/- 41 mg/dl. After controlling for other lipid parameters, demographics, and risk factors, the only cholesterol subfraction associated with carotid plaque was LDL (OR per standard deviation (SD) = 1.14, 95% CI 1.02-1.27). Neither HDL nor triglycerides independently predicted carotid plaque. Apolipoprotein B (ApoB) was also associated with risk of plaque (OR per SD = 1.29, 95% CI 1.03-1.60). Apolipoprotein A-I (apoA-1) was associated with a decrease in multiple plaques (OR per SD = 0.76, 95% CI 0.60-0.97), while lipoprotein a was associated with an increased risk of multiple plaques (OR per SD = 1.31, 95% CI 1.03-1.66). ApoB:ApoA-I had the strongest relation with carotid plaque (OR per SD = 1.35, 95% CI 1.08-1.69).

**Conclusions:**

Among the common lipid parameters, LDL has the strongest relation with carotid plaque. Other lipid precursor proteins such as ApoB and ApoA-I may be stronger predictors of subclinical atherosclerosis, however, and better targets for treatment to reduce plaque formation and risk of cerebrovascular disease.

## Background

Blood lipids levels, particularly elevated low-density lipoprotein (LDL) and decreased high-density lipoprotein (HDL), have been definitely associated with an increased risk of cardiovascular disease (CVD) and less clearly associated with stroke [1-4]. While there are several possible mechanisms underlying the association of lipids and stroke [5-7], one of the most important is probably the effects of lipids on the formation of carotid artery atherosclerotic plaque [8].

Although the protective effect of lipid-lowering medication on CVD and stroke risk is well-established [4,9,10], data regarding the relative importance of the various lipid parameters, total cholesterol (T-CHOL), LDL, HDL, non-HDL cholesterol, triglycerides (TG), lipoprotein a (Lp(a)), apolipoprotein (Apo) B and A-I, to CVD and stroke risk is inconclusive. A large prospective cohort study of women indicated a significantly elevated risk of first cardiovascular events, including ischemic stroke, among those at the extreme quintiles of all lipid parameters, including ApoA-I and ApoB, the major protein constituents of HDL and LDL, respectively. The most predictive parameters were non-HDL, HDL, and ApoB, and the lipid ratios were even more predictive, particularly T-CHOL:HDL [1]. Information regarding the association between lipid parameters and plaque formation is also limited, particularly in racially diverse populations.

While it has been suggested that ApoB and ApoA-I may play a more influential role in CVD prevention than total LDL and HDL, further research is needed to understand the optimal indicators of effective lipid-lowering treatment in patients at risk for stroke. The goal of this population-based study is to elucidate the relation between lipid levels and carotid plaque presence in a multi-ethnic cohort. Due to the complexity of lipid effects on atherosclerosis we hypothesize that the ratio of lipid parameters, rather than single lipid parameters, will be most associated with carotid plaque phenotypes. The association between lipid parameters, including lipid ratios, and various cardiovascular outcomes has been well-studied. However, research among Hispanic populations is lacking and is particularly important due to the growing population of Hispanics in the United States and the perception of evidence for a Hispanic paradox, which suggests that Hispanics have better cardiovascular health than what would be predicted based on their vascular risk factor profile.

## Methods

### Subjects

The Northern Manhattan Study (NOMAS) is a prospective, population-based, cohort study documenting stroke incidence, risk factors, and prognosis in a multiethnic urban community. Based in northern Manhattan, an area of approximately 260,000 people with 104,000 over age 39 in 1990, this study population has a unique race/ethnic distribution that is over 50% Hispanic. Methodology for NOMAS has been described previously [11].

A total of 3298 subjects were recruited and enrolled from 1993-2001. Eligible participants (1) had never been diagnosed with an ischemic stroke, (2) were ≥40 years old, and (3) resided for at least three months in a household with a telephone in northern Manhattan. Subjects were identified by random-digit dialing, and interviews were conducted by trained bilingual interviewers [12]. This study was approved by the University of Miami institutional review board, and written consent was obtained.

Subjects were recruited from the telephone sample to have an in-person baseline interview and assessment. The enrollment response rate was 75%. Standardized questions were adapted from the validated Centers for Disease Control and Prevention Behavioral Risk Factor Surveillance System [13,14].

### Exposure Assessment

Blood samples were drawn after an overnight fast. Plasma levels of cholesterol and triglycerides were measured using standardized enzymatic procedures with a Hitachi 705 automated spectrophotometer (Boehringer Mannheim, Mannheim, Germany). HDL was measured after precipitation of plasma apo B-containing lipoproteins with phosphotungstic acid. LDL was calculated using the Friedewald et al. equation [15]. The inter-assay coefficients of variation in our laboratory were 2% for T-CHOL, 4% for triglycerides, and 3% for HDL [16]. In addition to T-CHOL and its subfractions, serum levels of the LDL and HDL constituents, ApoB and ApoA-I, were also collected by commercially available immunonephelometric procedures. The interassay coefficients of variation for these measurements were 3-5% [17]. Lp(a) levels were determined using a rate nephelometric assay (Beckman Instruments, Brea, CA) adapted for use on a Beckman Array 360 nephelometer, as described previously [17,18]. Lp(a) antibody was obtained from Dako Chemicals (Carpinteria, CA), and Lp(a) standards were purchased from International Enzymes (Fallbrook, CA). These standards have been standardized against the Northwest Research Lipid Laboratories (Seattle, WA). This assay had coefficients of variation of 4% and 8% at Lp(a) levels of 48 and 8 mg/dl, respectively.

### Outcome Assessment

Carotid artery plaque presence was assessed by high-resolution B-mode ultrasound using a GE LogIQ 700 system with a multifrequency 9 to 13 MHz linear-array transducer. Carotid ultrasounds were performed according to standard scanning and reading protocols by trained and certified technologists as detailed previously [19-21]. Left and right carotid bifurcations and internal and common carotid arteries were examined for plaque presence. Plaque was defined as an area of focal wall thickening 50% greater than surrounding wall thickness confirmed by marking and comparing plaque thickness with that of the surrounding wall during scanning by electronic calipers. The plaque boundaries were traced manually from the digitized multiangled images. Maximum carotid plaque thickness (MCPT, millimeters) was measured at the peak plaque prominence from any of the three carotid artery segments using a semi-automatic IMAGE-Pro V.5.2 software (Microsoft). The intraclass correlation coefficients for within and between reader variability of plaque thickness measurements were 0.94 and 0.77, respectively (n = 88) [22]. Plaque surface characteristics (regular, irregular) were also recorded, and intra- and interrater correlations of plaque surface characteristics were greater than 0.90 in our laboratory.

The primary outcome of interest was any plaque presence, and secondary outcomes were (1) MCPT >1.9 mm, (2) more than one plaque in the carotid arteries, and (3) irregular plaque. Each of these outcomes was compared to the lack of plaque as the reference. A MCPT cutoff of 1.9 mm was used because it was previously shown to be predictive of cardiovascular events in this study population [23].

### Covariates

Covariates in multivariate analyses included demographic and vascular risk factors measured at baseline. Hypertension was defined as a self-reported history of hypertension or a measured systolic blood pressure ≥140 or diastolic blood pressure ≥90 mmHg. Diabetes was defined as self-report of such history or a fasting blood sugar ≥126 mg/dL. Body mass index (BMI) was measured continuously as kg/m^2^. Pipe and cigar smoking was assessed by self-report and categorized as current (within one year), former, and never smoker. Cigarette smoking was also assessed by self-report and defined in pack-years. Moderate alcohol use was defined as current drinking of >1 drink per month and ≤2 drinks per day. Leisure-time/recreation physical activity was assessed by a questionnaire [24]. Moderate to heavy physical activity level was defined as engaging in one or more of selected rigorous physical activities in a typical 14-day period. Race and ethnicity were defined by self-identification based on a series of interview questions modeled after the US census and was categorized as Hispanic, non-Hispanic white, and non-Hispanic black. Individuals of other race/ethnicity were excluded.

### Statistical Analyses

The following lipid levels were assessed continuously, as the primary exposures of interest: T-CHOL, HDL, LDL, TG, non-HDL (T-CHOL-HDL), ApoB, ApoA-I, and Lp(a). The following lipid ratios were also calculated and assessed continuously: T-CHOL:HDL, LDL:HDL, and ApoB:ApoA-I. Independent samples t-tests were conducted as univariate analyses to compare the lipid parameters among those with each of the plaque outcome criteria vs. those with no carotid plaque.

Multiple logistic regression analyses were conducted to examine the association between lipid parameters and plaque outcomes, controlling for other pertinent lipid parameters, demographics (age, sex, race/ethnicity), vascular and lifestyle risk factors (hypertension, diabetes, BMI, pipe or cigar smoking, pack-years of cigarette smoking, moderate alcohol use, moderate-to-heavy physical activity), and the change in time between lipid and plaque measurements.

Analyses of LDL, HDL, and triglycerides were mutually adjusted for each other; Non-HDL was adjusted for HDL; Lp(a) and ApoB were adjusted for HDL and triglycerides; ApoA-I was adjusted for LDL and triglycerides. For all lipid parameters the odds ratios (OR) and 95% confidence intervals (CI) are reported for a 1 standard deviation (SD) increase.

We explored potential heterogeneity by race/ethnicity, sex, and age in the association between total cholesterol and its subfractions with presence of plaque by adding interaction terms in the multivariate analyses.

The total number of individuals with measurements of both carotid plaque and T-CHOL and its subfractions (LDL, HDL, triglycerides) was 2341, from which 537 were excluded because their carotid ultrasounds were conducted more than a year prior to their blood lipid measurements. Therefore, the total sample size for this study was 1804, of which 568 had measurement of ApoB and ApoA-I and 582 had Lp(a). Only 3% of the study population had missing covariate data.

## Results

Plaque was present in 61% of participants (N = 1106), of whom 211 (19%) had irregular plaque, 727 (66%) had multiple plaques, and 514 (46%) had MCPT >1.9 mm. Plaque was observed most commonly in the internal carotid artery and bifurcation (N = 1093, 99% of those with plaque), and less frequently in the common carotid artery (N = 123, 11% of those with plaque).

Table 1 shows the characteristics of the study population, overall and stratified by plaque presence. Twenty-two percent had LDL <100, 33% had LDL 100-130, 27% 130-160, and 17% >160 mg/dl. Thirty-one percent had HDL levels <40, 32% had HDL between 40-50, and 36% >50 mg/dl. Only 13% of the study population reported taking lipid-lowering medication, as the baseline data were collected prior to the publication of the ATP III guidelines.

**Table 1:** Characteristics of the study population: overall and by plaque presence

	Overall (N = 1804)	No plaque (N = 698)	Plaque (N = 1106)
**Covariates**			
**Age (Mean ± SD)**	68.5 ± 10.1	64.5 ± 9.6	71.1 ± 9.5^*a*^
**Male sex (%)**	39.8	37.8	41.0
**Race/ethnicity**			
**Hispanic (%)**	51.4	61.6	44.9^*a*^
**Black (%)**	25.5	22.1	27.7^*a*^
**White (%)**	23.1	16.3	27.4^*a*^
**Hypertension (%)**	73.5	68.6	76.5^*a*^
**Diabetes (%)**	21.2	18.8	22.7^*a*^
**Cigarette smoker**			
**Current (%)**	15.5	12.2	17.6 ^*a*^
**Former (%)**	37.8	32.7	40.9 ^*a*^
**Pipe or cigar smoker**			
**Current (%)**	2.3	1.5	2.7^*a*^
**Former (%)**	5.9	4.4	6.8 ^*a*^
**Moderate alcohol consumption (%)**	35.1	34.7	35.3
**Moderate to heavy physical activity (%)**	10.0	12.0	8.8^*a*^
**BMI (Mean ± SD)**	27.9 ± 5.6	28.3 ± 5.6	27.7 ± 5.6^*a*^
**Lipid Parameters (Mean ± SD)**			
**Total cholesterol (N = 1804)**	202 ± 41	200 ± 40	203 ± 42
**LDL (N = 1804)**	127 ± 37	125 ± 37	128 ± 37
**HDL (N = 1804)**	48 ± 15	48 ± 15	48 ± 15
**Triglycerides (N = 1804)**	133 ± 71	135 ± 80	133 ± 65
**Non-HDL (N = 1804)**	154 ± 41	152 ± 40	155 ± 42
**Lp(a) (N = 582)**	41 ± 41	39 ± 36	43 ± 44
**ApoB (N = 568)**	113 ± 30	108 ± 29	116 ± 30^*a*^
**ApoA-I (N = 568)**	146 ± 28	147 ± 29	145 ± 27
**T-CHOL:HDL (N = 1804)**	4.58 ± 1.57	4.54 ± 1.52	4.60 ± 1.61
**LDL:HDL (N = 1804)**	2.92 ± 1.24	2.87 ± 1.18	2.95 ± 1.27
**ApoB:ApoA-I (N = 568)**	0.81 ± 0.29	0.77 ± 0.30	0.83 ± 0.28^*a*^

^*a *^p < 0.05 vs. no plaque, X^2 ^test for categorical variables and t-test for continuous variables

Tables 1 and 2 show the mean ± SD for each of the lipid parameters, stratified by plaque outcome criteria. Individuals with each plaque outcome had higher ApoB and ApoB:ApoA-I ratio than those with no plaque, and those with multiple plaques also had slightly elevated LDL.

**Table 2:** Mean and SD of lipid parameters, stratified by carotid plaque outcomes

Mean ± SD	Multiple plaques (N = 727)	Thickness >1.9 mm (N = 514)	Irregular plaque (N = 211)
**Total cholesterol**	203 ± 43	204 ± 41	203 ± 45
**LDL**	129 ± 38^*a*^	129 ± 36	129 ± 41
**HDL**	47 ± 15	48 ± 16	48 ± 15
**Triglycerides**	135 ± 67	131 ± 60	131 ± 65
**Non-HDL**	156 ± 43	155 ± 40	155 ± 47
**Lp(a)**	45 ± 45	44 ± 43	40 ± 33
**ApoB**	118 ± 29^*a*^	117 ± 31^*a*^	125 ± 36^*a*^
**ApoA-I**	144 ± 28	145 ± 28	143 ± 28
**T-CHOL:HDL**	4.68 ± 1.65	4.59 ± 1.60	4.57 ± 1.81
**LDL:HDL**	3.01 ± 1.30	2.95 ± 1.27	2.93 ± 1.45
**ApoB:ApoA-I**	0.85 ± 0.29^*a*^	0.84 ± 0.30^*a*^	0.91 ± 0.35^*a*^

^*a*^p < 0.05 vs. no plaque (t-test)

Table 3 shows the OR and 95% CI for the relation between each of the lipid parameters and each of the carotid plaque outcomes in the multivariate-adjusted models. Increased T-CHOL, LDL, non-HDL, ApoB, T-CHOL:HDL, LDL:HDL, and ApoB:ApoA-I were associated with plaque presence, whereas HDL, triglycerides, Lp(a), and ApoA-I were not. A 1-SD increase in T-CHOL (mg/dL) was associated with a 12% increased risk of plaque. Of the total cholesterol subfractions, only LDL was an independent predictor of plaque (OR for a 1-SD increase = 1.14). As triglycerides were not associated with plaque, LDL is driving the association with non-HDL (OR for a 1-SD increase in non-HDL = 1.13). The LDL precursor protein ApoB was even more predictive of plaque presence than LDL (OR for a 1-SD increase = 1.29).

**Table 3:** Association between lipid parameters and carotid plaque outcomes, adjusted for demographics and vascular risk factors^*a*^

	OR (95% CI)^*b *^vs. No plaque			
	Plaque	Multiple plaques	Thickness >1.9 mm	Irregular plaque
**Total cholesterol**	1.12 (1.01-1.25)	1.15 (1.02-1.30)	1.11 (0.97-1.27)	1.08 (0.91-1.28)
**LDL**	1.14 (1.02-1.27)	1.16 (1.03-1.31)	1.13 (0.99-1.29)	1.09 (0.91-1.29)
**HDL**	0.97 (0.86-1.09)	0.91 (0.79-1.05)	0.96 (0.83-1.11)	1.10 (0.90-1.33)
**Triglycerides**	0.99 (0.88-1.10)	1.04 (0.92-1.18)	1.01 (0.88-1.16)	0.97 (0.81-1.17)
**Non-HDL**	1.13 (1.01-1.26)	1.17 (1.03-1.33)	1.12 (0.98-1.28)	1.07 (0.90-1.28)
**Lp(a)**	1.21 (0.99-1.48)	1.31 (1.03-1.66)	1.22 (0.94-1.58)	1.05 (0.55-1.99)
				
**ApoB**	1.29 (1.03-1.60)	1.29 (1.00-1.66)	1.35 (1.04-1.75)	1.56 (0.95-2.56)
**ApoA-I**	0.85 (0.70-1.05)	0.76 (0.60-0.97)	0.80 (0.63-1.03)	0.83 (0.48-1.41)
**T-CHOL:HDL**	1.13 (1.01-1.26)	1.23 (1.08-1.40)	1.16 (1.01-1.33)	1.00 (0.83-1.19)
**LDL:HDL**	1.16 (1.04-1.29)	1.25 (1.10-1.41)	1.18 (1.03-1.36)	1.03 (0.86-1.23)
**ApoB:ApoA-I**	1.35 (1.08-1.69)	1.49 (1.15-1.94)	1.41 (1.08-1.84)	1.46 (1.06-2.01)

^*a *^age, sex, race/ethnicity, hypertension, diabetes, BMI, pack-years of cigarette smoking, pipe or cigar smoking (past/current/never), moderate alcohol use, moderate to heavy physical activity, and the change in time between lipid and plaque measurements

^*b *^1 SD increase

Although the ratios T-CHOL:HDL and LDL:HDL were positively associated with plaque, as well as the presence of multiple plaques and thick plaque, in the multivariate analyses, the ratio of precursor proteins ApoB:ApoA-I was far more predictive, with a 35% increased risk of plaque observed for a 1-SD increase in ApoB:ApoA-I.

When the models were further adjusted for lipid-lowering medication use, the conclusions remained unchanged (data not shown). Interaction terms were added to the multivariate models to examine potential effect modification by race/ethnicity, sex, and age, and no significant interactions were observed.

The relative risk estimates for T-CHOL, LDL, non-HDL, and LDL:HDL were attenuated when the outcome was restricted to irregular plaque (vs. no plaque), although the association with ApoB appeared stronger. In contrast, the lipid parameters were generally more strongly predictive when the outcome was restricted to the presence of multiple plaques. Individuals with multiple plaques had significantly elevated levels of Lp(a) and lower ApoA-I.

## Discussion

The results indicate a strong relation between blood lipid levels and presence of carotid plaque in our multi-ethnic population, suggesting that blood lipids likely influence the risk of cardiovascular diseases and particularly stroke due to their effects on atherosclerotic plaque formation. Elevated LDL was independently associated with carotid plaque. However, HDL and triglycerides were not significantly associated with plaque presence after controlling for other lipid parameters and vascular risk factors. An analysis of precursor proteins supported the strength of the LDL association, as ApoB, the LDL precursor protein, was associated with an increased risk of plaque, whereas ApoA-I, the HDL precursor protein, was not. In fact, the lipid parameter most predictive of plaque presence was ApoB, and particularly the ratio ApoB:ApoA-I.

Despite the lower prevalence of carotid plaque observed among the Hispanic participants, the effect of lipid levels on plaque risk was not different among the Hispanics in comparison to the other race/ethnic groups. Therefore, we did not find evidence in support of the Hispanic paradox [25] regarding the effect of blood lipids. Further research is needed on vascular risk factors for subclinical atherosclerosis in Hispanics, as Hispanics comprise the fastest growing minority population in the United States, and it is important for clinicians to be aware of possible race/ethnic differences in the etiology of vascular disease.

Although current clinical practices involve the measurement of T-CHOL, LDL, HDL, and triglycerides to inform treatment decisions, the results of the current study suggest that the precursor protein ApoB and, to a lesser extent, ApoA-I may be stronger predictors of subclinical atherosclerosis, and therefore may be helpful parameters to use in clinical practice. In fact, the ratio ApoB:ApoA-I may be the best predictor of risk of atherosclerosis and likewise the optimal measure to assess new lipid-lowering medication, as treatment regimens that lower ApoB are likely to be most effective in reducing plaque formation.

The atherosclerotic process is very complex and involves several different mechanisms including injury in the endothelium, activation of immuno-inflammatory cells, smooth muscle cell proliferation, and influx of lipoproteins through the vessel injury space [26,27]. The results of the current study do not suggest that HDL is unrelated to carotid plaque presence. Rather, they indicate that it is the relative levels of LDL and HDL that may be more predictive than either LDL or HDL alone, and that the HDL precursor protein ApoA-I may be more etiologically relevant than the overall HDL level. The anti-atherogenic property of HDL has been documented in both clinical observations and laboratory settings [28], as HDL has been shown to have both direct and indirect anti-inflammatory effects with potential antithrombotic results [29]. The main beneficial role of HDL is its capacity to reverse cholesterol transport, removing free cholesterol from the macrophage in the endothelium and returning it to the liver for excretion into bile, thereby protecting against atherosclerotic plaque formation [5].

The association between the ApoB:ApoA-I ratio and development of atherosclerosis [30,31] and cardiovascular disease [32] has been shown. ApoB assembles the precursor of very-low-density lipoprotein, a component of LDL, while ApoA-I is necessary for the metabolism of HDL. Modification of the ApoB:ApoA-I ratio may affect atherosclerotic plaque development [33]. Interestingly, it has been shown that, unlike lipoproteins LDL and HDL, the apolipoproteins remain stable with age and cardiovascular disease severity [34]. We did not show that Lp(a) was independently associated with carotid plaque presence, although it was predictive of the presence of multiple plaques, and therefore may be associated with a greater plaque burden. The restricted sample of NOMAS individuals with Lp(a) measured limited the statistical power of these analyses. Data on the etiologic importance of Lp(a) has been controversial, as Lp(a) has been more related to the mechanisms of thrombosis and fibrinolysis. A recent study showed that Lp(a) was independently associated with carotid stenosis and occlusion, but not with plaque area [35]. A substudy of 426 individuals selected from the NOMAS population previously showed that maximum internal carotid artery plaque thickness was not associated with Lp(a) levels, but it was associated with the amount of Lp(a) carrying the small apo(a) size [36].

We did not find that triglycerides were associated with plaque presence after controlling for other lipid parameters and vascular risk factors. It has been shown that triglycerides are the lipid subfraction least present inside atherosclerotic plaque [37]. Furthermore, the fact that triglyceride levels vary considerably within individuals makes it more difficult to evaluate them as a risk factor for atherosclerosis. Other studies have also demonstrated no significant association between triglycerides and carotid plaque after controlling for other lipid parameters and vascular risk factors [38,39].

Carotid plaque, particularly thick plaque and irregular plaque, has been shown to be a risk factor for vascular events, including stroke [23,40-42]. Because lipids likely influence the risk of CVD and stroke due to their effects on atherosclerotic plaque formation, carotid plaque may be used as a surrogate endpoint in epidemiologic and pharmacologic studies of lipid treatment.

The various lipid parameters and lipid ratios examined in our study have been shown to be predictive of combined cardiovascular events in a large population-based study of women in which the lipid parameters were not mutually adjusted for each other [1]. Interestingly, the strongest effect estimate was observed for non-HDL. This study showed that the lipid ratios were more predictive of cardiovascular disease risk than any of the individual lipid levels. However, unlike our study, the weakest association was observed for the ratio of ApoB:ApoA-I. Although lipid parameters have been well-established as predictors of coronary heart disease, their role in the pathogenesis of stroke has been more controversial. In the Atherosclerotic Risk in Communities (ARIC) study, investigators reported weak and inconsistent [nonlinear] associations between LDL, HDL, triglycerides, ApoB, and ApoA-I with ischemic stroke risk [43]. In contrast, two other large studies have demonstrated strong associations between T-CHOL and its subfractions with risk of ischemic stroke [44,45]. Lipid parameters have also been shown to be related to carotid intima-media thickness (IMT), a related yet distinct measure of subclinical atherosclerosis [34].

Studies examining the effects of lipid-lowering treatments have provided strong evidence for an important role of lipids, particularly LDL, in stroke risk. Two meta-analyses have suggested an 18% decreased risk of stroke among statin users as compared to controls [46,47]. The SPARCL study demonstrated a decreased risk of recurrent stroke in individuals treated with atorvastatin following a previous stroke or TIA [48]. A recent meta-analysis also suggested that the effect of statins on both stroke risk and carotid IMT progression observed across studies was closely associated with the reduction in LDL cholesterol [49].

Strengths of the current study include its large and ethnically-diverse sample population, including Hispanics, collection of information on correlated vascular risk factors necessary to estimate the independent association between lipids and carotid plaque, assessment of a range of lipid parameters, including Lp(a) which has been relatively unstudied, and the measurement of various plaque characteristics in addition to its simple presence (e.g. thickness, irregularity, number). However, a primary limitation is the cross-sectional nature of this study. Further research in other populations is necessary to examine the association between lipid levels ascertained longitudinally and the progression of carotid plaque, in order to better understand the temporality of the relation. In addition, information on Lp(a), ApoB, and ApoA-I were available for a subsample, limiting the statistical power with which to examine these less traditional lipid parameters.

## Conclusions

In conclusion, our study supports the role of total cholesterol, LDL, and particularly the precursor protein ApoB as predictors of carotid atherosclerotic plaque in a multi-ethnic population, independent of other traditional vascular risk factors. Results suggest that ApoB may be the best target for treatment in people at risk for atherosclerosis and cerebrovascular disease. The current study provides novel information on the relative utility of various lipid parameters in predicting carotid plaque, an important subclinical marker of atherosclerosis, and contributes to the growing evidence for the superior prognostic value of the apolipoproteins.

## Competing interests

The authors declare that they have no competing interests.

## Authors' contributions

HG performed the statistical analysis and drafted the manuscript. DM participated in the drafting of the manuscript. MSVE participated in the design of the study and helped to draft the manuscript. TR participated in the design of the study and helped to draft the manuscript. RLS conceived of the study, participated in its design, and helped to draft the manuscript. All authors read and approved the final manuscript.

## Pre-publication history

The pre-publication history for this paper can be accessed here:

http://www.biomedcentral.com/1471-2261/9/55/prepub
